# Outcomes of emergent hiatal hernia repair: does specialty matter?

**DOI:** 10.1007/s00464-026-13287-2

**Published:** 2026-08-24

**Authors:** Khaled Noueihed, Jacob Woodroof, George Ibrahim, Scott Koeneman, John Jacob, Tyler R. Grenda, Nathaniel R. Evans, Olugbenga T. Okusanya

**Affiliations:** 1 Division of Esophageal and Thoracic Surgery, Department of Surgery, Thomas Jefferson University Hospital, 1015 Walnut Street, Curtis Building, Suite 613, Philadelphia, PA 19107 USA; 2 Division of Biostatistics and Bioinformatics, Thomas Jefferson University, Philadelphia, PA 19107 USA

**Keywords:** Hiatal hernia, Emergency surgery, Specialty, Paraesophageal hernia, Outcomes

## Abstract

**Background:**

Hiatal hernia repair is a common procedure performed by both general and thoracic surgeons. While prior studies have shown that emergent hiatal hernia repairs are associated with worse outcomes than elective cases, differences in outcomes by surgical specialty for emergent repairs have not been well-described.

**Methods:**

The American College of Surgeons–National Surgical Quality Improvement Program (ACS–NSQIP) database was queried for patients undergoing hiatal hernia repair from 2012 to 2024. Patients undergoing elective repair and non-primary repairs were excluded. Perioperative outcomes were compared between patients with procedures performed by general and thoracic surgeons. Multivariable logistic regression was used to evaluate differences in outcomes after adjustment for demographics, comorbidities, and preoperative risk factors.

**Results:**

A total of 2,629 patients were identified, including 2,443 (92.9%) performed by general surgery and 186 (7.1%) performed by thoracic surgery. The majority of cases were performed laparoscopically (74.9%) with no mesh placement (70%). On unadjusted analysis, perioperative outcomes were similar between groups. After multivariable adjustment, surgical specialty was not independently associated with 30-day mortality (OR 0.67, 95% CI 0.26–1.73), readmission (OR 0.81, 95% CI 0.45–1.44), reoperation (OR 1.64, 95% CI 0.89–3.04), overall morbidity (OR 1.16, 95% CI 0.79–1.70), and length of stay (β = 0.15 days, 95% CI –1.05 to 1.36).

**Conclusions:**

Among patients undergoing emergent hiatal hernia repair, there were no significant differences in short-term outcomes between general and thoracic surgeons after multivariable adjustment.

**Graphical abstract:**

**Figure:**
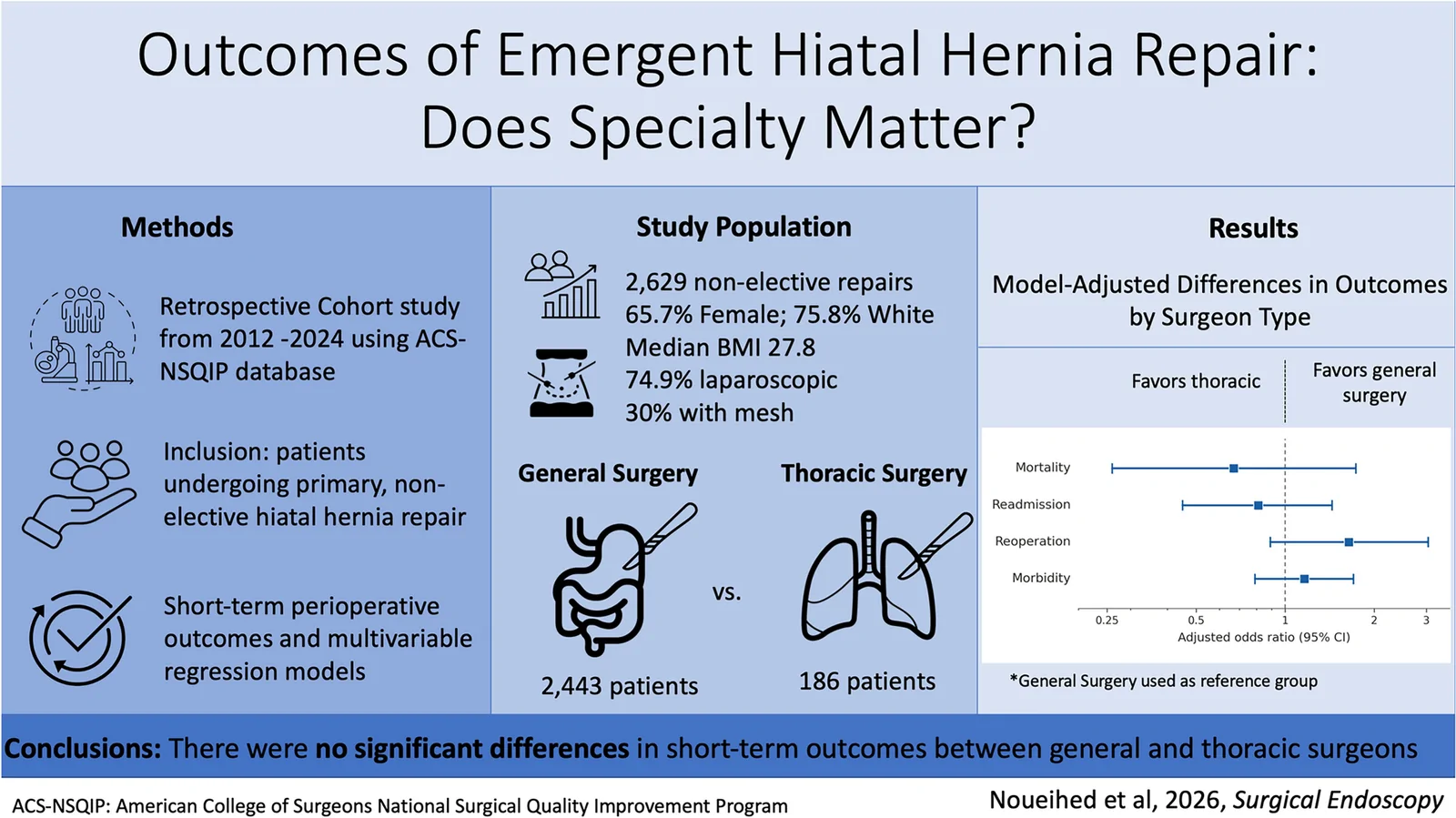

**Supplementary Information:**

The online version contains supplementary material available at https://doi.org/10.1007/s00464-026-13287-2.

Hiatal hernias are a common disease process estimated to occur in about 10–20% of the population [1, 2]. Symptoms can widely range, including heartburn, dysphagia, regurgitation, chest pain, early satiety, and more, which may delay diagnosis [3–7]. Management depends on the severity and type of the hernia, with surgery typically reserved for patients with reflux who fail medical management, severe esophagitis, or large, symptomatic hernias [8–10]. While most cases are managed electively, a subset of patients develop acute complications, such as incarceration, gastric volvulus, perforation, cardiopulmonary decompensation, and hemorrhage, necessitating an emergent operation [11, 12]. The annual probability of developing acute symptoms requiring emergency surgery in patients with hiatal hernia is estimated to be around 1% [12]. Prior literature has shown that patients undergoing emergent repair were associated with worse outcomes than those undergoing elective hiatal hernia repair [13–16].

Hiatal hernia repair is an operation performed by both general and thoracic surgeons. Prior studies have evaluated the impact of surgeon characteristics, such as volume and specialty, on outcomes across various procedures, with some evidence pointing that these characteristics may be associated with worse outcomes [17, 18]. In the context of hiatal hernia repair, existing literature has largely focused on elective cases, with prior studies suggesting no significant difference in outcomes based on surgeon specialty [19]. However, emergent hiatal hernia repair represents a fundamentally different clinical scenario, characterized by higher acuity and increased operative risk, and it remains unclear whether surgeon specialty influences outcomes in this setting.

This study utilizes a large, national cohort from the American College of Surgeons–National Surgical Quality Improvement Program (ACS–NSQIP) database to evaluate short-term outcomes following non-elective hiatal hernia repair and to compare outcomes between general surgeons and thoracic surgeons.

## Materials and methods

### Study population

This study was a retrospective cohort study utilizing the ACS–NSQIP database from 2012 to 2024. We included all patients undergoing urgent or emergent hiatal hernia repair from 2012 to 2024. Patients undergoing hiatal hernia repair were identified using Current Procedural Terminology (CPT) codes, including laparoscopic and open approaches, abdominal and thoracic approaches, and codes with and without mesh use. The exact CPT codes that were utilized were included in Supplemental Table 1. Patients undergoing elective repair and non-primary repairs were excluded. The NSQIP database allows for several CPT codes to be entered for a patient’s operation. In order to minimize bias, we only included patients who had hiatal hernia repairs as their primary operation. In addition, if a repair was performed by a specialty other than general surgery or thoracic surgery, those cases were excluded.

The reporting of this study conforms to the STROBE statement.

### Data source

The ACS–NSQIP is a large national database that collects data from participating hospitals. It contains over 150 variables, including demographics, comorbidities, surgical history, and short-term clinical outcomes. The American College of Surgeons National Surgical Quality Improvement Program and the hospitals participating in the ACS NSQIP are the source of the data used herein; they have not verified and are not responsible for the statistical validity of the data analysis or the conclusions derived by the authors. This research was deemed exempt from Institutional Review Board approval at Thomas Jefferson University given the database was de-identified.

### Demographic and clinical characteristics

Demographics of interest included age, body mass index (BMI), sex, race, specialty, functional status, ASA score, year of operation, and comorbidities including diabetes, severe COPD (as defined by ACS–NSQIP), hypertension, heart failure, immunosuppressive therapy, smoking and ventilator dependence.

Other relevant clinical information included operative indication and surgical acuity. The primary International Classification of Diseases (ICD) diagnosis code was extracted for each case as a proxy for the acute operative indication. Clinically relevant acute indications are reported individually; less frequent or non-specific diagnoses were combined into an "Other/Unspecified" category (Supplemental Table 2). Surgical acuity was defined using the ACS–NSQIP case acuity variable. NSQIP revised this variable during the study period: prior to 2021, acuity was recorded as a binary "Emergency" field (Yes/No), whereas from 2021 onward cases were classified as elective, urgent, or emergent. Because the earlier schema did not distinguish urgent from emergent cases, all pre-2021 emergency cases were classified as "Not Specified" to avoid retrospective reclassification.

Perioperative outcomes were compared between patients with procedures performed by general and thoracic surgeons. Outcomes of interest included 30-day mortality, 30-day readmission, reoperation, morbidity, and length of stay. Morbidity was defined as the presence of any of the following postoperative complications: pneumonia, reintubation, failure to wean from the ventilator within 48 h, superficial SSI (surgical site infection), deep SSI, organ-space SSI, wound disruption/dehiscence, pulmonary embolism, acute renal failure, urinary tract infection (UTI), stroke, cardiac arrest, myocardial infarction, bleeding requiring transfusions, deep venous thrombosis, sepsis, and septic shock.

Multivariable logistic regression was used to evaluate differences in outcomes between surgeon types after adjustment for confounders, including age (non-linear), body mass index (non-linear), functional status, history of diabetes, smoking, and anatomic approach. Anatomic approach stratified patients into laparoscopic, open abdominal (laparotomy), open chest (thoracotomy), and combined open chest and abdomen (thoracoabdominal approach). This characterization further distinguishes the thoracic from the abdominal approach while accounting for minimally invasive surgery. There is no CPT code for thoracoscopic hiatal hernia repair, precluding a separate minimally invasive thoracic category. Mesh placement was not included as a covariate, as mesh-specific morbidities (e.g., erosion, recurrence) are not captured by ACS–NSQIP and typically manifest beyond its 30-day window. Linear regression was used to evaluate differences in length of stay among surgeon type while also adjusting for the abovementioned covariates. Wald-type confidence intervals were constructed for model effects to better evaluate estimated differences between surgeon types. During model creation, the general surgery group was used as the reference group.

All analyses were performed using R Statistical Software (v4.3.1, R Core Team 2025).

## Results

**Fig. 1:**
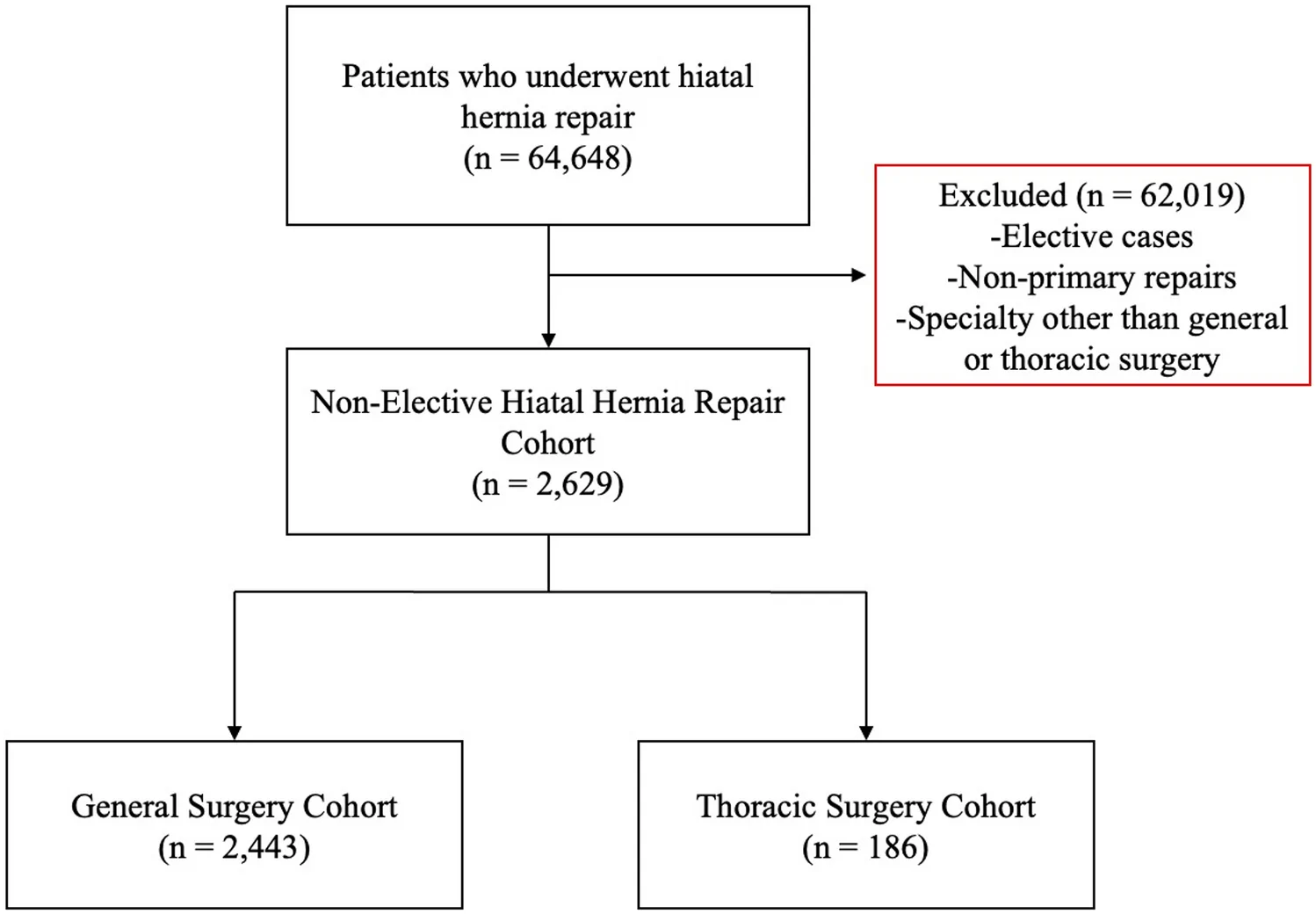
Inclusion criteria diagram

**Fig. 2:**
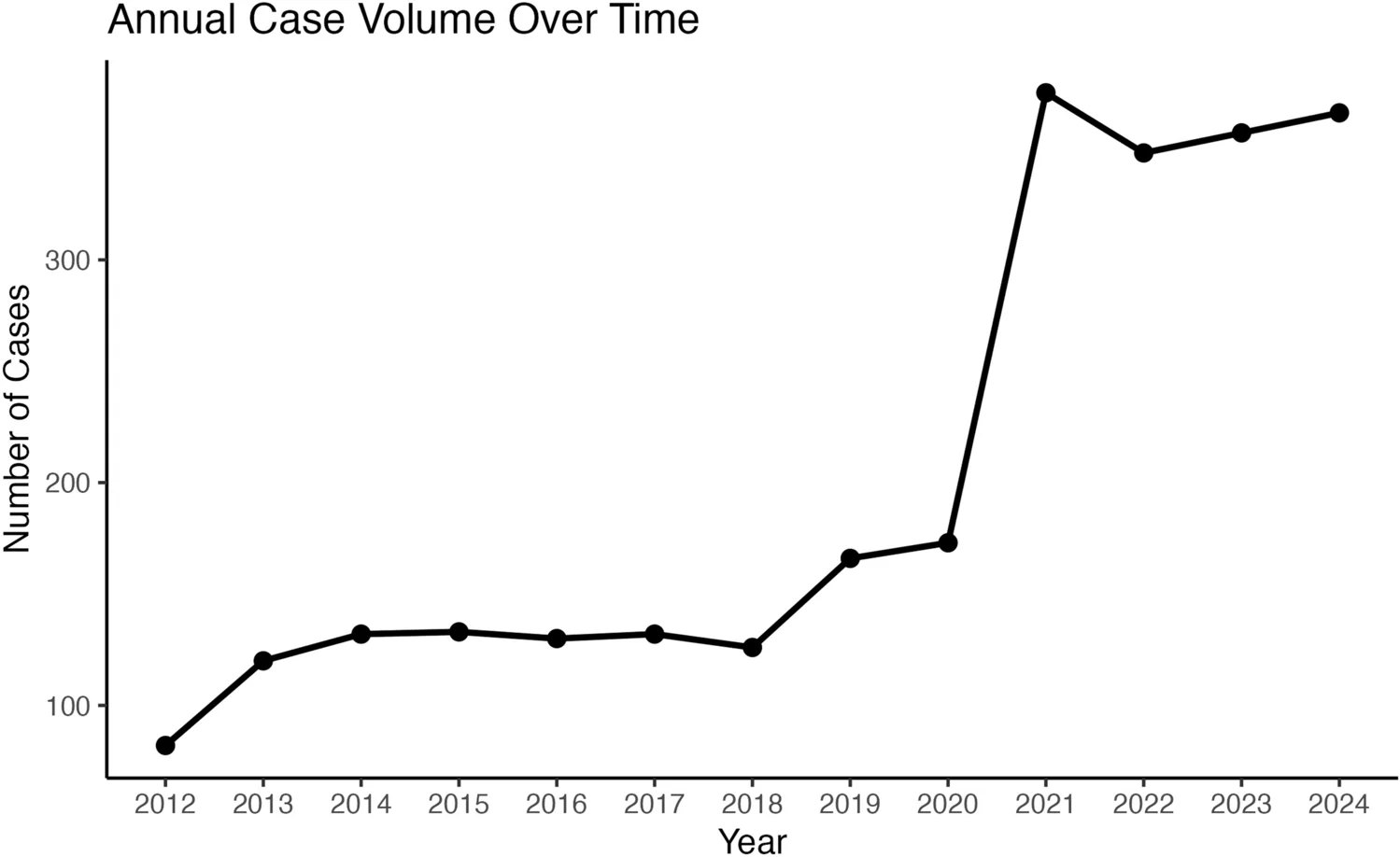
Annual non-elective hiatal hernia repair case volume over study period

A total of 64,648 patients underwent hiatal hernia repair from 2012 to 2024. Of these, 2,629 patients underwent primary hiatal hernia repair in an urgent or emergent setting (Fig. 1). The majority of cases (92.9%, *n* = 2,443) were performed by general surgeons, with 7.1% (*n* = 186) performed by thoracic surgeons. The median age of the full cohort was 72 years (IQR 60–81), with a median BMI of 27.8 (IQR 24.2–31.9). Most patients were female (65.7%) and White (75.8%). The majority were functionally independent (89.9%) with an ASA score of 3–5 (76.9%). Most cases were performed laparoscopically (74.9%) and without mesh (70%) (Table 1). After laparoscopy, laparotomy was the second most common repair approach (*n* = 568, 21.6%). Evaluating temporal trends, annual case volume remained relatively stable from 2012 to 2020, followed by a marked increase from 2021 to 2024 (Fig. 2).

**Table 1:** Demographics and clinical variables of full cohort

Variable	Subcategory	Full cohort (*n* = 2,629)
Continuous variables	Age (years)	72 (60–81)
	BMI (kg/m^2^)	27.8 (24.2–31.9)
	LOS (days)	6 (3–9)
Sex	Female	1,727 (65.7%)
	Male	902 (34.3%)
Race	White	1,993 (75.8%)
	Black	147 (5.6%)
	Asian	24 (0.9%)
	Other^†^	55 (2.1%)
	Unknown	410 (15.6%)
Hispanic ethnicity	Yes	201 (7.6%)
	No	2,073 (78.9%)
Specialty	General surgery	2,443 (92.9%)
	Thoracic	186 (7.1%)
Functional status	Independent	2,363 (89.9%)
	Partially independent	184 (7%)
	Totally dependent	39 (1.48%)
ASA score	1–2	606 (23.1%)
	3–5	2,019 (76.9%)
Year	2012	82 (3.1%)
	2013	120 (4.6%)
	2014	132 (5%)
	2015	133 (5.1%)
	2016	130 (4.9%)
	2017	132 (5%)
	2018	126 (4.8%)
	2019	166 (6.3%)
	2020	173 (6.6%)
	2021	375 (14.3%)
	2022	348 (13.2%)
	2023	357 (13.6%)
	2024	366 (13.9%)
Case priority	Urgent	890 (33.9%)
	Emergent	554 (21.1%)
	Not specified*	1,185 (45.1%)
Comorbidities	Diabetes	280 (10.6%)
	Severe COPD	177 (6.7%)
	Hypertension	1,490 (56.7%)
	Heart failure	101 (3.8%)
	Immunosuppressive therapy	161 (6.1%)
	Smoking	218 (8.3%)
	Ventilator dependent	51 (1.9%)
Operative approach	Open	660 (25.1%)
	Laparoscopic	1,969 (74.9%)
Anatomic approach	Laparoscopic	1,969 (74.9%)
	Open abdominal (laparotomy)	568 (21.6%)
	Open chest (thoracotomy)	41 (1.6%)
	Open chest and abdomen (thoracoabdominal approach)	51 (1.94%)
Mesh placement	Yes	790 (30%)
	No	1,839 (70%)

Data presented as *N* (%) or median (IQR)

*ASA* American Society of Anesthesiologists, *BMI* body mass index, *COPD* chronic obstructive pulmonary disease, *LOS* length of stay

^†^Other race included American Indian or Alaska Native, Hawaiian or Pacific Islander, and other unspecified race

^*^Not specified case priority represents emergency cases that were not further defined as Urgent or Emergent

After performing analysis for the surgical specialty, demographics of the general surgery cohort were largely similar to the full cohort, given that it comprises a large majority of it. Patients in the thoracic surgery cohort were slightly younger (median 70 vs. 72) with similar BMI (27.5 vs. 27.8). Similarly, most patients in the thoracic cohort were female (61.8%) and White (43%, *n* = 80), but a substantial proportion had unknown or unreported race (51.1%). Thoracic surgeons were less likely to perform laparoscopic repair (60.8% vs. 76.0%) and less likely to place mesh (19.4% vs. 30.9%) (Table 2). The primary ICD-coded operative indication, stratified by specialty, is reported in Supplemental Table 2. Among general surgery cases, diaphragmatic hernia with obstruction was the most common indication, whereas thoracic cases were more evenly divided between obstruction and diaphragmatic hernia without a specified acute modifier. Cases performed for diaphragmatic hernias with gangrene, ulcers, and pyloric stenosis were only performed by general surgeons. Case priority and operative year, stratified by specialty, are reported in Supplemental Table 3; the specialty composition of the cohort remained stable across the study period, with general surgery performing the majority of cases across all years.

**Table 2:** Demographics and clinical variables by specialty

Variable	Subcategory	General surgery (*n* = 2,443)	Thoracic surgery (*n* = 186)
Continuous variables	Age (years)	72 (60–81)	70 (61–79)
	BMI (kg/m^2^)	27.8 (24.2–31.9)	27.5 (24.5–31.8)
	LOS (days)	6 (3–9)	7 (4–10)
Sex	Female	1,612 (66%)	115 (61.8%)
	Male	831 (34%)	71 (38.2%)
Race	White	1,913 (78.3%)	80 (43%)
	Black	138 (5.6%)	9 (4.8%)
	Asian	23 (1%)	1 (0.5%)
	Other^†^	54 (2.2%)	1 (0.5%)
	Unknown	315 (12.9%)	95 (51.1%)
Hispanic ethnicity	Yes	193 (7.9%)	8 (4.3%)
	No	1,987 (81.3%)	86 (46.2%)
Functional status	Independent	2,190 (89.6%)	173 (93%)
	Partially independent	175 (7.2%)	9 (4.8%)
	Totally dependent	37 (1.5%)	2 (1.1%)
ASA score	1–2	566 (23.2%)	40 (21.5%)
	3–5	1,873 (76.8%)	146 (78.5%)
Year	2012	76 (3.1%)	6 (3.2%)
	2013	115 (4.7%)	4 (2.2%)
	2014	125 (5.1%)	7 (3.8%)
	2015	128 (5.2%)	4 (2.2%)
	2016	123 (5%)	5 (2.7%)
	2017	126 (5.2%)	4 (2.2%)
	2018	116 (4.7%)	9 (4.8%)
	2019	151 (6.2%)	15 (8.1%)
	2020	160 (6.5%)	13 (7%)
	2021	347 (14.2%)	27 (14.5%)
	2022	322 (13.2%)	26 (14%)
	2023	327 (13.4%)	30 (16.1%)
	2024	327 (13.4%)	36 (19.4%)
Case priority	Urgent	804 (32.9%)	86 (46.2%)
	Emergent	520 (21.3%)	34 (18.3%)
	Not specified*	1,119 (45.8%)	66 (35.5%)
Comorbidities	Diabetes	258 (10.6%)	22 (11.8%)
	Severe COPD	156 (6.4%)	21 (11.3%)
	Hypertension	1,395 (57.1%)	95 (51.1%)
	Heart failure	94 (3.8%)	7 (3.8%)
	Immunosuppressive therapy	145 (5.9%)	16 (8.6%)
	Smoking	197 (8.1%)	21 (11.3%)
	Ventilator dependent	46 (1.8%)	5 (2.7%)
Operative approach	Open	587 (24%)	73 (39.2%)
	Laparoscopic	1,856 (76%)	113 (60.8%)
Anatomic approach	Laparoscopic	1,856 (76%)	113 (60.8%)
	Open abdominal (laparotomy)	527 (21.6%)	41 (22%)
	Open chest (thoracotomy)	20 (0.8%)	21 (11.3%)
	Open chest and abdomen (thoracoabdominal approach)	40 (1.6%)	11 (5.9%)
Mesh placement	Yes	754 (30.9%)	36 (19.4%)
	No	1,689 (69.1%)	150 (80.6%)

Data presented as *N* (%) or median (IQR)

*ASA* American Society of Anesthesiologists, *BMI* body mass index, *COPD* chronic obstructive pulmonary disease, *LOS* length of stay

^†^Other race included American Indian or Alaska Native, Hawaiian or Pacific Islander, and other unspecified race

^*^Not specified case priority represents emergency cases that were not further defined as Urgent or Emergent

Evaluating unadjusted outcomes between groups, compared with general surgery cases, patients operated by thoracic surgeons experienced lower 30-day mortality (3.8% vs. 4.8%, OR 0.77, 95% CI 0.3–1.67), and 30-day readmission (7.5% vs. 10.5%, OR 0.7, 95% CI 0.37–1.22) (Table 3). The thoracic surgery group had higher rates of reoperation (8.1% vs. 5.3%, OR 1.56, 95% CI 0.83–2.75), similar overall morbidity (24.7% vs. 24.4%, OR 1.02 (0.7–1.45), and a longer median length of stay (7 vs. 6 days) compared to the general surgery group.

**Table 3:** Unadjusted postoperative outcomes following emergent hiatal hernia repair by specialty

Variable	Full (*n* = 2,629)	General surgery (*n* = 2,443)	Thoracic surgery (*n* = 186)	OR (95% CI)*
30-day mortality	125 (4.8%)	118 (4.8%)	7 (3.8%)	0.77 (0.3–1.67)
30-day readmission	270 (10.3%)	256 (10.5%)	14 (7.5%)	0.7 (0.37–1.22)
Reoperation	145 (5.5%)	130 (5.3%)	15 (8.1%)	1.56 (0.83–2.75)
Morbidity	641 (24.4%)	595 (24.4%)	46 (24.7%)	1.02 (0.7–1.45)

^*^Odds ratios use General Surgery as the reference group

Data are presented as *N* (%)

After multivariable adjustment, surgical specialty was not independently associated with 30-day mortality (OR 0.67, 95% CI 0.26–1.73), readmission (OR 0.81, 95% CI 0.45–1.44), reoperation (OR 1.64, 95% CI 0.89–3.04), overall morbidity (OR 1.16, 95% CI 0.79–1.70), and length of stay (β = 0.15 days, 95% CI –1.05 to 1.36), with the general surgery cohort as the reference group (Table 4).

**Table 4:** Model-adjusted outcomes following emergent hiatal hernia repair by specialty

Variable	Effect estimate (95% CI)*
Length of stay (days)	+ 0.15 days (−1.05–1.36)
30-day mortality	0.67 (0.26–1.74)
30-day readmission	0.81 (0.45–1.48)
Reoperation	1.64 (0.89–3.04)
Morbidity	1.16 (0.79–1.70)

^*^Effect estimates represent odds ratios for categorical outcomes and mean differences (in days) for length of stay. General surgery was used as the reference group

## Discussion

We performed a national analysis evaluating short-term outcomes following non-elective hiatal hernia repair between general and thoracic surgeons. We found that surgical specialty was not independently associated with mortality, readmission, reoperation, morbidity, or length of stay after multivariable adjustment. These findings suggest that, in the emergent setting, outcomes may be driven more by patient factors and disease acuity than by surgeon specialty.

Prior studies have described worse outcomes for emergent hiatal hernia repairs compared to elective cases. Jassim et al. reported significantly higher morbidity and mortality among patients undergoing emergent hiatal hernia repair compared to elective repairs [15], highlighting the high-risk nature of this population. This study matches Jassim’s findings on demographics: the majority of patients undergoing emergent repair were female and White, though their cohort was younger (65.5 vs. 72) than ours, and only 26.9% had a laparoscopic repair, compared with 74.9% in ours. This reflects differences in contemporary practice, with a higher proportion of laparoscopic repairs likely due advancement in minimally invasive techniques with broader surgeon adoption over time. Their described morbidity rate was higher (33.4% vs. 24.4%), but their described mortality rate was lower (3.2% vs. 4.8%) than our findings. Direct comparison of morbidity is limited by differences across databases, arbitrary definitions, and variable capture. The higher mortality observed in our cohort likely reflects differences in patient comorbidity burden or case severity. This reinforces the high-risk nature of emergent hiatal hernia repair and suggests that patient factors, rather than approach or specialty, may be the dominant drivers of outcomes in this population.

The impact of surgeon characteristics, particularly specialty and volume, on postoperative outcomes has been widely studied across surgical disciplines. A common procedure of choice, carotid endarterectomy, has been extensively studied and may have some similarities to our population, as this is a procedure that can be performed by vascular surgeons, general surgeons, cardiothoracic surgeons, and neurosurgeons. Several studies have evaluated differences in outcomes by specialty. Although findings are mixed, most studies conclude that outcomes do not differ significantly by specialty and are more strongly associated with volume and indication, with high-volume surgeons and non-emergent cases associated with better post-operative outcomes [17, 18, 20]. Although we were unable to evaluate surgeon volume, our findings are consistent with this broader literature, suggesting that specialty alone is not a primary determinant of outcomes in high-acuity settings.

Looking further into the intersection of hiatal hernia repair outcomes and surgeon specialty, Hadaya et al. conducted a study evaluating patients undergoing hiatal hernia repair and comparing outcomes between general and thoracic surgeons using the NSQIP database from 2012 to 2019. Their study concluded that surgical specialty was not associated with mortality or major complications [19]. Their findings reflected that functional status and severity of underlying medical illness were more strongly associated with poorer outcomes. This study builds on their findings by including a longer study period and focusing only on emergent cases. Patients undergoing emergent hiatal hernia repair represent an older and frailer population that face increased perioperative risk, extended hospital stays, and worse morbidity [15, 21, 22]. In this context, the absence of an outcome difference by specialty suggests that specialty designation alone may be less important than timely operative intervention, although our data cannot speak to the contributions of surgeon volume or individual experience.

We also observed a marked increase in non-elective cases after 2020. This pattern is most likely explained, at least partially, by a change in ACS–NSQIP acuity coding during the study period. Prior to 2021, acuity was recorded as a binary "Emergency" variable (Yes/No); from 2021 onward, cases were classified as elective, urgent, or emergent. The introduction of a distinct urgent category allowed capture of acute presentations that the earlier binary variable did not separately identify; emergent case counts after 2021 were comparable to the annual "Not Specified" volumes recorded beforehand (Supplemental Table 3), supporting the interpretation that the rise largely reflects newly distinguishable urgent cases rather than a true increase in emergent presentations. This trend may also reflect delayed care during the COVID-19 pandemic, leading to the postponement of elective repairs and potentially increasing rates of acute presentation. However, the concurrent coding transition limits our ability to attribute the trend to any single cause.

These findings have important clinical implications. In emergent hiatal hernia repair, where delays in care may worsen outcomes, timely operative intervention by an available and capable surgeon may be more critical than specialty designation alone. Institutional workflows should prioritize expeditious surgical management, and both general and thoracic surgeons may be reasonable providers in this setting. Efforts to improve outcomes should instead focus on optimizing patient selection, perioperative management, and system-level factors such as access to care and hospital resources.

This study has several limitations. First, we are severely limited by our cohort size, particularly the thoracic surgery cohort. Given that only 186 cases met the inclusion criteria for the thoracic group, there were not enough thoracic-associated events to perform a more robust analysis, as reflected by the width of the confidence intervals. Importantly, we did not perform formal non-inferiority or equivalence testing; the absence of a statistically significant difference by specialty should, therefore, not be interpreted as evidence of equivalence between groups. We are additionally limited by our database. The NSQIP database is a retrospective database with inherent selection bias, and a limited variable list. We are not able to have the desired granularity to describe relevant outcomes, such as prior surgical history, recurrence, or longer postoperative outcomes. Surgeon volume and experience may play an important role in surgical outcomes; unfortunately, the ACS–NSQIP database does not record surgeon- or institution-level identifiers, case volume, or other markers of individual experience. This is particularly relevant given the learning curve associated with laparoscopic hiatal hernia repair, which predominates in our cohort. As such, we were unable to adjust for these factors in our analysis, which is an additional limitation of the study. Furthermore, we are not able to further identify subspecialty training within the general surgery group. The general surgery group may include minimally invasive or acute care surgery-trained physicians, which we are not able to report. In addition, we are unable to elucidate whether patients had been previously evaluated for hiatal hernia repair through the NSQIP data. It is possible that a subset of patients had been evaluated for hiatal hernia repair but were deemed too high risk to proceed with surgery. This would in part explain the poorer outcomes noted in the emergent group. However, the worse outcomes may be confounded by poor surgical candidacy rather than the emergent nature of the disease process, which we cannot differentiate. Furthermore, the database does not capture important anatomical or technical characteristics of the hernias — such as the degree of intrathoracic migration, proximity to mediastinal vasculature, prior repairs, specific acute complications prompting surgery, or other markers of complexity — and we were, therefore, unable to stratify outcomes by hernia complexity. NSQIP is only able to capture data from affiliated institutions, which limits the generalizability of this study. Finally, our analysis was dependent on CPT codes, so coding inconsistencies may influence reported data.

In conclusion, we did not detect a significant difference in short-term outcomes between general and thoracic surgeons among patients undergoing non-elective hiatal hernia repair. Most emergent hiatal hernia repairs are performed laparoscopically without mesh placement. Elective repairs by experienced surgeons remain a better option when feasible. Efforts to improve outcomes should focus on expediting repair and optimizing perioperative care when emergent repair is unavoidable. Future studies should prioritize volume-outcome analyses that incorporate surgeon and institutional volume, as well as other markers of operative experience, which are strong determinants of outcomes in this population.

## Supplementary Information

Below is the link to the electronic supplementary material.

Supplementary file1 (DOCX 12 KB)

Supplementary file2 (DOCX 14 KB)

Supplementary file3 (DOCX 16 KB)
