# Supplementary material for: Outcomes of emergent hiatal hernia repair: does specialty matter?
Source: Surg Endosc. 2026 Aug 24;40(11):9886–94. doi: 10.1007/s00464-026-13287-2 (PMC13755106; doi:10.1007/s00464-026-13287-2)
Supplement: Supplementary file 1 [file 464_2026_13287_MOESM1_ESM.docx]

Supplemental Table 1. Current Procedural Terminology (CPT) codes for Hiatal Hernia Repair

| Operation | CPT Code |
| --- | --- |
| Laparoscopic Repair with Mesh | 43282 |
| Laparoscopic Repair without Mesh | 43281 |
| Open Repair with Mesh | 43333 (abdominal), 43335 (thoracotomy), 43337 (thoracoabdominal) |
| Open Repair without Mesh | 43332 (abdominal), 43334 (thoracotomy), 43336 (thoracoabdominal) |
