# Supplementary material for: Outcomes of emergent hiatal hernia repair: does specialty matter?
Source: Surg Endosc. 2026 Aug 24;40(11):9886–94. doi: 10.1007/s00464-026-13287-2 (PMC13755106; doi:10.1007/s00464-026-13287-2)
Supplement: Supplementary file 2 [file 464_2026_13287_MOESM2_ESM.docx]

Supplemental Table 2. Primary ICD-coded operative indication for non-elective hiatal hernia repair, overall and by surgical specialty

| **Case Description** | **Total** | **General Surgery (%)** | **Thoracic Surgery (%)** |
| --- | --- | --- | --- |
| Diaphragmatic hernia with obstruction | 1205 | 1128 (93.6%) | 77 (6.4%) |
| Diaphragmatic hernia with gangrene | 34 | 34 (100%) | 0 (0%) |
| Diaphragmatic hernia  without specified obstruction or gangrene | 829 | 748 (90.2%) | 81 (9.8%) |
| Volvulus | 70 | 65 (92.9%) | 5 (7.1%) |
| Ulcer | 28 | 28 (100%) | 0 (0%) |
| Adult hypertrophic pyloric stenosis | 42 | 42 (100%) | 0 (0%) |
| Other diseases of stomach and duodenum | 145 | 142 (97.9%) | 3 (2.1%) |
| Other/Unspecified* | 276 | 256 (92.8%) | 20 (7.2%) |

Operative indication was approximated using the primary ICD diagnosis code recorded in ACS-NSQIP. Percentages represent the proportion of cases within each indication performed by general versus thoracic surgeons.

*Other/Unspecified Category included less frequent or non-specific diagnoses.

ICD: International Classification of Diseases.
