# Supplementary material for: Outcomes of emergent hiatal hernia repair: does specialty matter?
Source: Surg Endosc. 2026 Aug 24;40(11):9886–94. doi: 10.1007/s00464-026-13287-2 (PMC13755106; doi:10.1007/s00464-026-13287-2)
Supplement: Supplementary file 3 [file 464_2026_13287_MOESM3_ESM.docx]

Supplemental Table 3. Case priority by operative year and surgical specialty

| **Case Year** | **Case Type** | **Total** | **General Surgery Cases (%)** | **Thoracic Surgery Cases (%)** |
| --- | --- | --- | --- | --- |
| 2012 | Not Specified | 80 | 74 (92.5%) | 6 (7.5%) |
| 2013 | Not Specified | 120 | 116 (96.7%) | 4 (3.3%) |
| 2014 | Not Specified | 133 | 126 (94.7%) | 7 (5.3%) |
| 2015 | Not Specified | 130 | 126 (96.9%) | 4 (3.1%) |
| 2016 | Not Specified | 130 | 125 (96.2%) | 5 (3.8%) |
| 2017 | Not Specified | 128 | 124 (96.9%) | 4 (3.1%) |
| 2018 | Not Specified | 125 | 116 (92.8%) | 9 (7.2%) |
| 2019 | Not Specified | 165 | 151 (91.5%) | 14 (8.5%) |
| 2020 | Not Specified | 174 | 161 (92.5%) | 13 (7.5%) |
| 2021 | Urgent | 222 | 205 (92.3%) | 17 (7.7%) |
|  | Emergent | 153 | 143 (93.5%) | 10 (6.5%) |
| 2022 | Urgent | 235 | 214 (91.1%) | 21 (8.9%) |
|  | Emergent | 114 | 108 (94.7%) | 6 (5.3%) |
| 2023 | Urgent | 218 | 197 (90.4%) | 21 (9.6%) |
|  | Emergent | 139 | 130 (93.5%) | 9 (6.5%) |
| 2024 | Urgent | 215 | 188 (87.4%) | 27 (12.6%) |
|  | Emergent | 148 | 139 (93.9%) | 9 (6.1%) |

“Not Specified” Case Priority represents cases recorded under the legacy ACS-NSQIP binary "Emergency" variable (Yes/No), which did not distinguish urgent from emergent cases. Beginning in 2021, ACS-NSQIP classified acuity into elective, urgent, and emergent categories; cases from 2021 onward are reported using these designations.
